# Comparison of Guided and Unguided Ocriplasmin Injection for the Treatment of Vitreomacular Traction: A Preliminary Study

**DOI:** 10.1155/2016/6521304

**Published:** 2016-03-15

**Authors:** Rodolfo Mastropasqua, Luca Di Antonio, Vincenzo Ciciarelli, Agbeanda Aharrh-Gnama, Marco Rispoli, Paolo Carpineto

**Affiliations:** ^1^Ophthalmology Unit, Department of Neurological, Neuropsychological, Morphological and Movement Sciences, University of Verona, 37100 Verona, Italy; ^2^Ophthalmology Clinic, Department of Medicine and Aging Science, University “G. d'Annunzio” of Chieti-Pescara, 66100 Chieti, Italy; ^3^Centro Oftalmologico Mediterraneo, 00195 Roma, Italy

## Abstract

This retrospective quality control study aimed at comparing resolution in patients treated with intravitreal ocriplasmin (IVO) using two injection techniques, classical injection procedure (unguided) and targeted injection using a surgical microscope with a 30-gauge 1-inch needle (guided) for the treatment of focal VMT without macular hole. The two groups presented a statistically significant difference in terms of resolution of VMT within the first month following treatment: 1/7 for the unguided group versus 6/7 for the guided group (*p* = 0.0291). The majority of the guided group presented an earlier resolution than the single resolved case in the unguided group. The results of this preliminary study indicate that the injection of ocriplasmin closer to the site of VMT results in the resolution in a higher number of cases and that this resolution occurs in a short time interval.

## 1. Introduction

The scaffold of the vitreous is composed of collagen fibers (mostly type II) and hyaluronic acid and the posterior vitreous cortex is a complex structure. In the aging eye, after age 40, the vitreous gel begins to liquefy involving nearly 50% by age 80, a process called synchysis. During this time the posterior vitreous scaffold normally detaches from the retina with fluid filling the intervening space.

Vitreomacular traction (VMT) occurs when the vitreous separates from the retina throughout the peripheral fundus but remains adherent posteriorly, causing an anteroposterior traction on a small region encompassing the macular and optic nerve disc. Symptoms include vision impairment and metamorphopsia, accompanied by photopsia. VMT syndrome is associated with a broad spectrum of maculopathies, including cystoid macular edemas, epiretinal membranes (ERM), and macular holes (MH) [1–3].

Current treatment options include intravitreal injections with pharmacological agents that induce an enzymatic vitreolysis to induce complete VMT resolution. One of these pharmacological agents, ocriplasmin (Jetrea, Thrombogenics, Leuven, Belgium), was approved in October 2012 by the United States Food and Drug Administration for the treatment of symptomatic VMT [4]. It was reported to have a superior efficacy for VMT resolution compared to placebo injection in phase III randomized, controlled trials [4].

Although intravitreal injections are considered a cornerstone of retinal care and one of the most commonly performed procedures across all specialties, the technique is still undergoing evolution. Currently, the principle debates concerning intravitreal injections concern the gauge of the needle and the angle/path of scleral penetration [5, 6]. An important aspect of this procedure that has not been adequately evaluated concerns the site in which the agents are released.

For over twenty years, forensic pathologists have known the importance of extracting all of the vitreous humor for analysis given that it presents variations in solute concentration if sampled in the center or in periphery [7]. Filas et al. reported that in vivo vitreolytic agents cause the vitreous to contract and to lose its capacity to bind water with digestion. They also found that proteins spanning the vitreoretinal interface are not affected by hyaluronidase and concluded that these combined effects could exacerbate traction. This could explain why transient vision loss is observed after intravitreal injections [8]. de Smet et al. also recommended injecting the enzyme close to the VMT in order to improve effectiveness [9]. Based on these results we decided to modify our injection procedure by performing a standard intravitreal injection procedure with a surgical microscope equipped with an OCT scanner in an operating room setting and a longer needle in order to release the pharmaceutical agent closer to the VMT.

This retrospective quality control study aimed at comparing resolution in patients treated with intravitreal ocriplasmin (IVO) using two different injecting techniques, standard injection procedure (unguided) and proximal injection using a surgical microscope and a 30-gauge 1-inch needle (guided) for the treatment of focal VMT without macular hole (MH).

## 2. Patients and Methods

This quality assessment [10] nonrandomized retrospective consecutive case series study adhered to the tenets of the Declaration of Helsinki and received approval from an institution review board. The first fourteen consecutive patients with VMT without MH treated with intravitreal ocriplasmin with two different injecting techniques between June 2014 and September 2015 at the Ophthalmic Clinic of University Chieti-Pescara (Italy) were enrolled. Informed consent was obtained from all patients prior to their enrollment. Patients underwent complete ophthalmic evaluation, including LogMAR visual acuity (VA) testing, and spectral-domain optical coherence tomography (SD-OCT) at baseline and follow-up controls.

### 2.1. Outcome Measures

The primary outcome measure was VMT resolution. Secondary outcomes were time to resolution, visual acuity, and central macular thickness measured with OCT.

Following injection, patients were visited one day after injection, and at five-day intervals for 30 days. They were also instructed to present within 24 hours if they noted a modification in their vision. During the first follow-up visit the treated eye was uncovered, VA was assessed, and an OCT was acquired. Visual acuity was assessed with the Early Treatment Diabetic Retinopathy Study (ETDRS) chart and standardized measurement criteria [11].

SD-OCT images were acquired through a dilated pupil using an RTVUE XR Avanti (version 2015.100.0.35, Optovue Inc., Fremont, CA) and RTVue-100 (Optovue version 5.1.0.90, Fremont, CA). Each evaluation was performed with both instruments and included a cross-line (10.00 mm scan length) and a 5 mm × 5 mm retinal map for central macular thickness (CMT). In addition, RTVUE XR Avanti was used to obtain enhanced HR line 12 mm scan length. Scans were evaluated by a single experienced ophthalmologist for the presence of VMT (vitreous adhesion within six mm of the fovea and elevation of the posterior vitreous cortex), for VMT release (defined as vitreous release from the macula within a six mm central retinal field), and for the absence of MH.

### 2.2. Intravitreal Injection Techniques

**Figure 1:**
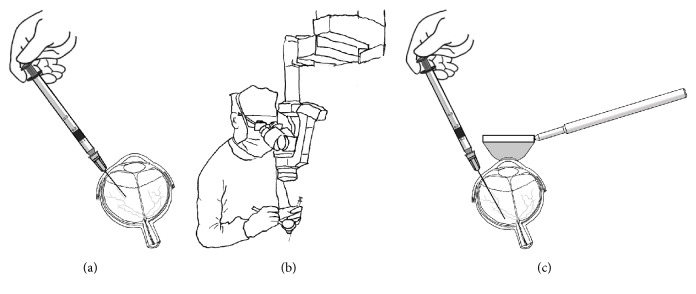
(a) The unguided injection techniques as suggested by the manufacturer. (b) Additional equipment (30-gauge 1-inch long needle, surgical microscope, and direct contact lens) used for the guided injections. (c) Detail of the injection site release which was not less than 3 mm.

The unguided standard injection procedure (unguided; Figure 1(a)) was performed according to the manufacturer's instructions (http://jetrea.com/wp-content/uploads/JETREAPrescribingInformation.pdf). Briefly, after the content of the vial reached room temperature, it was diluted with 0.2 mL of 0.9% w/v sodium chloride (sterile, preservative-free) using an aseptic technique. Then 0.1 mL (equivalent of 0.125 mg of ocriplasmin) was injected in the mid-vitreous area using a 30-gauge 1/2-inch injection needle, inserted 3.5–4.0 mm posterior to the limbus and aimed towards the center of the vitreous cavity, avoiding the horizontal meridian. The injections were performed in a sterile operating room but without using a surgical microscope.

The guided injections (Figures 1(b) and 1(c)) were performed in a similar manner except a 30-gauge 1-inch needle was used for the injection. Also, a surgical microscope and a contact retinal lens were used to determine the depth of insertion in order to guarantee that the area of release corresponded to the site of VMT (not less than 3 mm from the retinal plane). An intraoperative spectral-domain OCT system integrated into a surgical microscope (Rescan 700 iOCT: Zeiss, Oberkochen, Germany) and evaluation of the shadow produced by the needle on the retinal surface were used to determine the distance from the retina during intravitreal injection.

### 2.3. Statistical Analysis

All statistical analyses were performed using SPSS 22 (IBM, Armonk, NY) and evaluated at an alpha level of 0.05. Differences in baseline parameters were evaluated with Fisher's Exact Test (qualitative parameters) and two-tailed independent sample *t*-test with correction for results of Levene's Test for Equality of Variances (quantitative parameters). The primary outcome was evaluated with Fisher's Exact Test. The statistical significance of differences in the temporal distribution of VMT resolution was evaluated using Kaplan-Meier Survival Curves. Statistically significant variations of VA and CMT within group between time points and between groups with single time points were evaluated using two-tailed pair *t*-tests and Mann-Whitney Tests, respectively.

## 3. Results

All patients successfully completed follow-up. The two groups of seven patients did not present statistically significant differences in terms of age, sex, phakic lens status, and best corrected visual acuity (Table 1).

**Figure 2:**
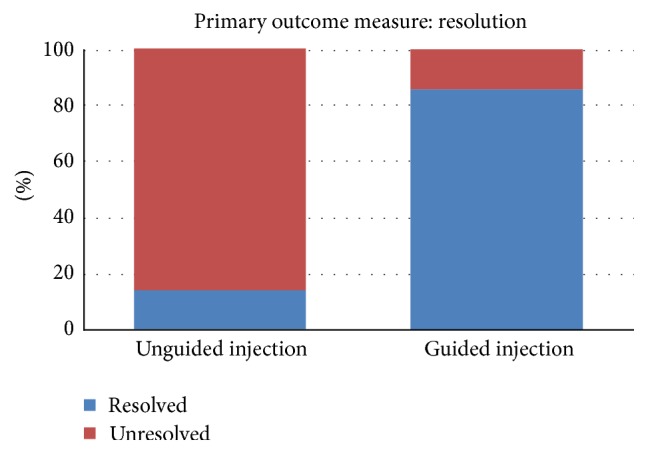
Distribution of patients presenting resolution of VMT within the first month following injection.

**Figure 3:**
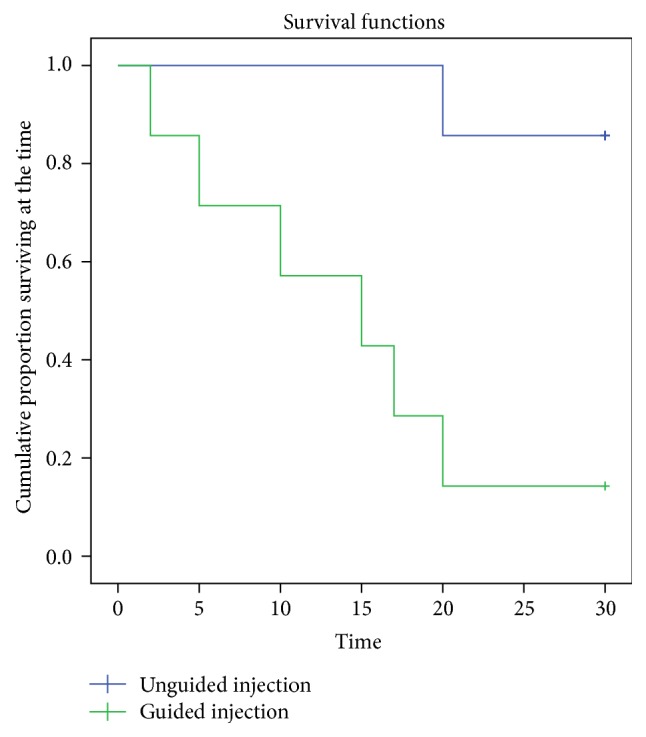
Kaplan-Meier Survival Curves of the temporal distribution of VMT resolution.

**Figure 4:**
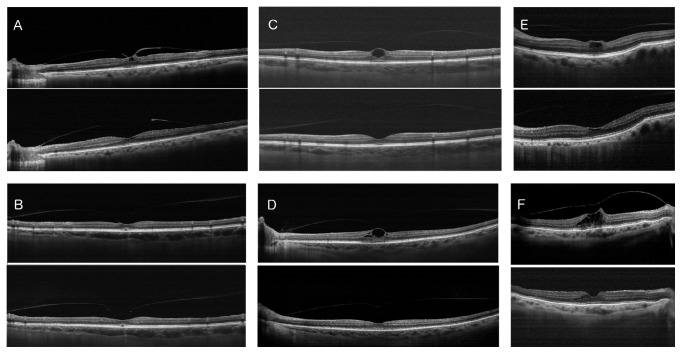
Cross-sectional optical coherence tomography images showing patients (from A to F) before (top) and after (bottom) resolution of vitreomacular traction after intravitreal guided injection of ocriplasmin.

The two groups presented a statistically significant difference in the main outcome measure (resolution of VMT) within the first month following treatment: 1/7 for the unguided injection group versus 6/7 for the guided injection group (*p* = 0.0291, Fisher's Exact Test, Figure 2). The Kaplan-Meier Survival Curve analysis of the temporal distribution of VMT resolution indicated a statistically significant difference between the two groups (*p* = 0.004, Figure 3). The majority of the guided injection group (Figure 4) presented an earlier resolution than the single resolved case in the unguided injection group.

**Figure 5:**
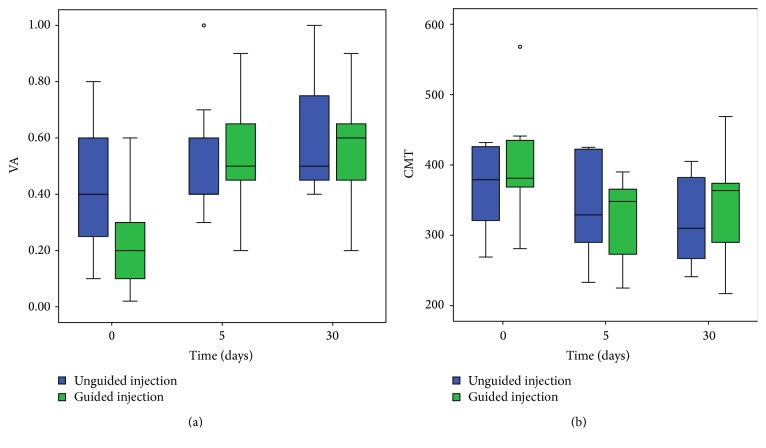
Box plot of visual acuity and central macular thickness. Intergroup differences at the three time points are not statistically significant.

Statistically significant intergroup differences for VA and CMT at the three time points were not observed (Figure 5). When the intrapatient variation was evaluated, statistically significant differences in VA were observed between baseline and five and thirty days for the unguided group and between baseline and five and thirty days for the guided group (Table 2).

Statistically significant differences in CMT were observed between baseline and thirty days for the unguided group and between baseline and five and thirty days for the guided group (Table 2).

## 4. Discussion

In this study we investigated the efficacy of ocriplasmin for VMT when using two different injecting procedures. The resolution of VMT was observed in 1/7 of patients in the unguided injection group and in 6/7 of patients in the guided injection group. In phase III clinical trials, the resolution of VMT after ocriplasmin injection was reported to be 26.5% ranging from 41% to 75% with higher percentages in patients with age less than 65 years, focal adhesions less than or equal to 1500 mm, phakic lens status, and absence of epiretinal membrane [12–14].

Previous studies reported comparable percentages of VMT resolution ranging from 42.1% to 66.7% [13, 15]. In our case series, patients did not differ significantly for baseline characteristics such as age (14/14 were more than 65-year-old), sex distribution (6/7 and 5/7 were female in the unguided and guided group, resp.), type of VMT (focal in 14/14 of eyes without ERM and/or MH), and lens status (pseudophakic in 14/14 patients); thus these aspects could not account for the differences between the two groups in resolution rate.

It was demonstrated that injected ocriplasmin has a high autolytic activity in vitreous and that the presence of partially liquefied vitreous may reduce the rate of autolysis prolonging the enzyme activity [9]. During aging the vitreous physiologically liquefies and separates from the retina; thus it is possible to hypothesize that, by placing the enzyme deep inside the vitreous cavity close to the site of VMT, increasing the probability that it will be in an area that has already undergone liquefaction, it will result in a high concentration and a prolonged action of the enzyme, accounting for the quicker and higher percentage of resolution in the ocriplasmin guided injection group.

The lower percentage of resolution in the ocriplasmin unguided injection group compared to clinical trials and real life studies was probably due to the small sample size rather than a real difference in the study population [13, 14].

The anatomic resolution of VMT was related to a significant CMT decrease with a mean percentage variation of 85% from 0 to 5 days (*p* < 0.04) and of 66% from 0 to 30 days (*p* < 0.005) in the guided group compared to a mean percentage variation of 23% from 0 to 5 (*p* < 0.251) and 46% from 0 to 30 days (*p* < 0.045) in the unguided group. The related increase of VA showed a mean variation of 0.3 logMAR from 0 to 5 and 0 to 30 days (*p* < 0.03) in the guided group compared to a mean variation of 0.1 decimal in the unguided group from 0 to 5 (*p* < 0.11) and of 0.2 from 0 to 30 days (*p* < 0.011).

Other authors demonstrated a similar improvement of macular anatomy after ocriplasmin injection. Chatziralli et al. described a decrease of macular thickness from 389 ± 152 *μ*m to 263 ± 99 *μ*m for cases with VMT release [15].

Favorable results in terms of visual acuity increase in the guided group were comparable to VA increase observed in phase III clinical trial showing at six months: a gain of two or more lines in 23.7% of patients treated with ocriplasmin compared to 11.2% of patients in the placebo group (*p* < 0.001) [12]. Sharma et al. observed an improvement of two or more lines at one month of follow-up and a gain of three lines or more for a mean follow-up of 258 days in patients treated with ocriplasmin for VMT [16].

This study presents several limits. Adverse events were not evaluated in this study given the low incidence with which they occur and the small sample size. Transitory vision loss was not evaluated since treated eyes were patched for the first 24 hours. Another limit of this study was the sample size. Therefore, prior to drawing conclusive results, a similar study should be performed in a larger study population using a multicenter randomized case-control design.

The results of this preliminary study indicate that the injection of ocriplasmin closer to the site of VMT results in the resolution in a higher number of cases and that this resolution occurs in a shorter time interval compared to a standard injection technique.

## Figures and Tables

**Table 1:** Comparison of group characteristics at baseline.

	Unguided	Guided	*p*
Age (years)	71.7 ± 10.3	73.1 ± 10.2	0.799^*∗*^
Male, *n* (%)	1/7	2/7	1.000^*∗*^
Adhesion size, maximum length (*μ*m)	293.27 ± 59.06	326.75 ± 48.02	0.267^*∗∗*^
Epiretinal membrane (presence)	2/7	2/7	1.000^*∗*^
Phakic lens status, *n* (%)	7/7	6/7	1.000^*∗*^
BCVA baseline (logMAR)	0.43 ± 0.26	0.23 ± 0.22	0.121^*∗∗*^
Central macular thickness (*μ*m)	368 ± 79	405 ± 89	0.406^*∗∗*^

^*∗*^Fisher's Exact Test; ^*∗∗*^two-tailed independent sample *t*-test.

**Table 2:** Secondary outcome measurements: Group mean of the modifications in visual acuity (logMAR) and central macular thickness (*μ*m) of individual patients between time points.

	Time (days)					
	0 versus 5 days		0 versus 30 days		5 versus 30 days	
VA (logMAR)	Mean variation	*p*	Mean variation	*p*	Mean variation	*p*

Unguided injection	0.1	0.111	0.2	0.011	0.1	0.045
Guided injection	0.3	0.003	0.3	0.003	0.3	0.356

CMT (*μ*m)	Mean percentage variation	*p*	Mean percentage variation	*p*	Mean percentage variation	*p*

Unguided injection	23	0.251	46	0.045	48	0.293
Guided injection	85	0.004	66	0.005	−20	0.218

*p*: two-tailed pair *t*-test.
